# Comparison of thromboprophylaxis patterns in arthroplasty in public and private hospitals

**DOI:** 10.1590/S1679-45082015GS3057

**Published:** 2015

**Authors:** Aline Pinheiro dos Santos Cortada, Telma Gomes da Silva, André Campos da Silva, Ricardo Prado Golmia, Renata Leborato Guerra, Maíra Libertad Soligo Takemoto, Roberta Dyonisio Canaveira Monteiro, Morton Aaron Scheinberg

**Affiliations:** 1 Associação de Assistência à Criança Deficiente, São Paulo, SP, Brazil.; 2 Hospital Israelita Albert Einstein, São Paulo, SP, Brazil.; 3 ANOVA Consultoria em Saúde, Rio de Janeiro, RJ, Brazil.; 4 Bristol-Myers Squibb, São Paulo, SP, Brazil.

**Keywords:** Venous thromboembolism/therapy, Arthroplasty, replacement, Knee/economy, Arthroplasty, replacement, hip/economics, Health systems/economics; Hospital, public, Hospitals, private

## Abstract

**Objective:**

To compare therapy for prophylaxis of venous thromboembolism and costs related to hospitalization of patients undergoing total knee and hip replacement within the context of the Brazilian health system.

**Methods:**

A retrospective study of patients undergoing arthroplasty in 2010 in a public hospital and two private hospitals in the state of São Paulo, conducted by means of medical record review. Costs were estimated based on the use of health care resources during hospitalization. A descriptive analysis was performed using frequency and mean (standard deviation) according to the type of care delivered (by public or private organization).

**Results:**

A total of 215 patients were evaluated, and 56.3% were submitted to knee surgery and 43.7%, to hip replacement. Approximately 88% and 98% of patients from public and private health services, respectively, received some form of venous thromboembolism prophylaxis, and enoxaparin was the drug most widely used in both systems. The total cost of prophylaxis was R$ 1,873.01 (R$ 26.38 per patient) in the public service and R$ 21,559.73 (R$ 163.33 per patient) in the private service. For the individuals who presented with thromboembolism, the average cost of hospitalization was R$ 6,210.80 and R$ 43,792.59 per patient in public and private health services, respectively.

**Conclusion:**

Thromboembolism prophylaxis in patients undergoing arthroplasty is most commonly used in the private health services than public organizations, despite its high costs in both services. The cost per patient with thrombosis during hospitalization was higher than the total cost of prophylaxis, suggesting that prevention is associated to better cost-benefit ratio.

## INTRODUCTION

Venous thromboembolism (VTE) is condition characterized by deep venous thrombosis (DVT) with the possibility of evolving to pulmonary embolism (PE).^1,2^ Some surgical procedures, such as total knee arthroplasty (TKA) and total hip arthroplasty (THA), represent an important factor associated with the occurrence of VTE, increasing the risk of its occurrence by 40 to 70%. In these cases, it is possible to adopt preventive measures, which make VTE the primary cause of avoidable in-hospital death.

Thromboprophylaxis can be performed with drug and non-drug strategies.^3^ Some studies demonstrated that, in several countries, including Brazil, the main strategy utilized for thromboprophylaxis is pharmacological, with non-fractioned and low-molecular-weight heparin as the primary anticoagulants used.^4^ However, a study performed in a large hospital in Brazil, in 2007, evaluated the treatment for thromboprophylaxis as inappropriate in 47% of cases, when compared to the 8th Guidelines for VTE Prophylaxis by the American College of Chest Physicians. Absence of thromboprophylaxis was the major reason for not being appropriate.^5^

The occurrence of VTE in patient submitted to TKA and THA may present with direct consequences in morbidity of patients and hospital expenses. DVT and PE can postpone hospital discharge by approximately 5 and 7 days, respectively, and are the main cause of hospital readmission after THA.^6^In the United States, one year after a VTE episode, 5.3% of patients required new hospitalization with VTE as a primary cause, and 14.3% as a secondary cause.^7^

Data on the VTE prophylaxis patterns and costs associated with care of patients submitted to TKA or THA operations in Brazil are limited.^8^ This study was developed with the purpose of filling this gap of local data. In this way, the objective of this study was to describe the VTE prophylaxis patterns and the costs during the hospital stay of patients submitted to TKA and THA, within the context of the Brazilian healthcare system.

## OBJECTIVE

To compare patterns of thromboprophylaxis in the public and private Brazilian healthcare systems.

## METHODS

An observational study with retrospective cohort, carried out in three hospitals–in that, two represent the private healthcare system (*Hospital da Associação deAssistência à Criança Deficiente* − AACD and *Hospital Santa Cruz*, both in the city of São Paulo − SP) and one the public healthcare system (*Hospital Mário Covas da Faculdade de Medicina do ABC, *in Santo André − SP). The study included patients with a minimum age of 18 years submitted to elective surgery for THA or TKA between January 1st, 2010 and December 31st, 2010, and who were not previously using antithrombotic therapy.

Information on the sociodemographic, clinical and surgical characteristics of patients, as well as on the use of thromboprophylaxis was retrospectively collected from patient´s medical record review. For the purpose of this study, confirmed VTE was defined as a report, in the medical chart, of the confirmation of a clinical suspicion of the event by the physician, besides the positive report of some diagnostic method (Doppler ultrasonography of vessels, helical computed tomography and pulmonary arteriography). In order to estimate the costs associated with admission of patients, information was collected as to types and quantity of resources in health used, such as medications, medical devices (prostheses), supplementary tests, expense of days spent in the ward and at the intensive care unit (ICU), and other treatments. The unit values of each type of resource were in accordance with the financial source, using the following official sources for pricing of healthcare resources: *Banco de Preços em Saúde do Ministério da Saúde do Brasil,* version 2008;^11^ *Lista de Conformidade da Câmara de Regulação do Mercado de Medicamentos* (CMED) *daAgência Nacional de Vigilância Sanitária* (ANVISA);^12^ *Sistema de Gerenciamento da Table de Procedimentos* (SIGTAP);^13^ 4th edition of the *Classificação Brasileira Hierarquizada de Procedimentos Médicos* (CBHPM);^14^ *Programa de Estudos Avançados em Administração Hospitalar e de Sistemas de Saúde do Hospital das Clínicas da Faculdade de Medicina da Universidade de São Paulo e da Fundação Getúlio Vargas* (PROAHSA 41 E 50).^15^

A descriptive analysis was made of the sample characteristics, in which the quantitative variables were expressed as mean and standard deviation, with dispersion measurement, and the qualitative variables, by frequencies and percentages. Total cost and mean cost per patient were estimated as per segmentation of care received by the patient (surgical procedure and hospital follow-up – including the costs of prophylaxis for VTE during hospitalization). The results were stratified considering the type of operation performed (TKA and THA), the type of healthcare system (Unified Health System – *Sistema Único de Saúde*, SUS and healthcare insurance companies - *Saúde Suplemtar* - SS) and according to the suspicion or occurrence of VTE. All analyses were made using the statistical software Stata, version MP11, and the R Project, version 2.13.1.

The study was approved by the Research Ethics Committee of the AACD, under number 44/2011.

## RESULTS

**Figure 1:**
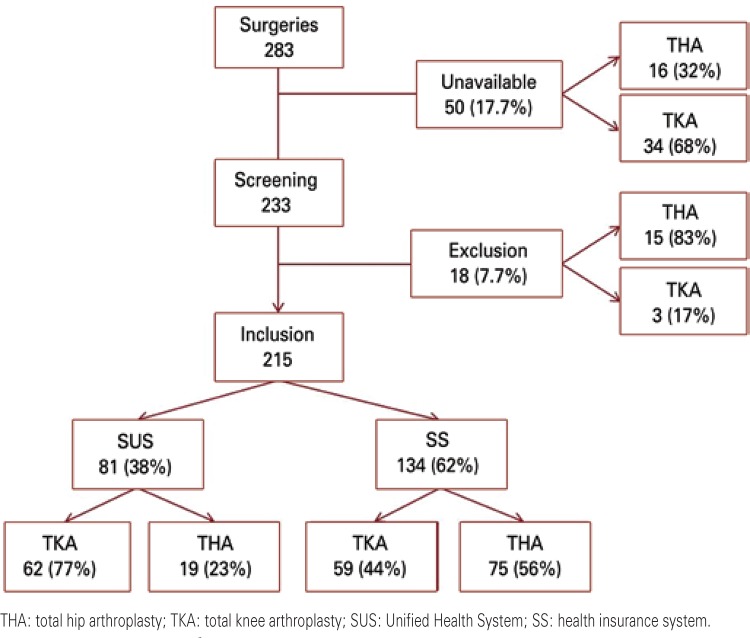
Flowchart of the study population

A total of 283 patients were submitted to TKA or THA at the selected centers, during the year 2010. Nevertheless, the medical records of 50 patients (17.7%) were not available for consultation. Among the patients with information available, 92.3% (215/233) were included in the study and 7.7% (18/233) were excluded for not satisfying the eligibility criteria. Among the patients evaluated in the study, 38% (81/215) came from the SUS (public system) and 62% (134/215) from the SS (health insurance system). Figure 1 shows the flowchart of the study population.

Approximately 56% (121/215) of patients in the sample were submitted to TKA and 44% (94/215) to THA, with primary arthroplasty in 80.2% (97/121) and 75.5% (71/94), and unilateral procedure in 97.5% (118/121) and 100% (94/94) of patients, respectively. The mean duration of the TKA operation was 2 hours and 55 minutes, with mean length of hospital stay of 5 days; whereas for THA, these means were, respectively, of 3 hours and 27 minutes, and 6 days. A greater frequency of bleeding as a complication was observed in THA (11.7% *versus* 1.7%).

The sample characteristics studied are described on table 1. Patients submitted to TKA presented with a mean age of 68.2 years (SD=9.8) and about 75% were females, while the patients submitted to the THA presented with mean age of 56.3 years (SD=15.6) and 53% were females. Most of the patients submitted to both operations were not smokers, were overweight, with mean body mass index (BMI) of 29.5kg/m^2^ for TKA, and 27.7kg/m^2^ for THA. The patients submitted to TKA had more comorbidities than those undergoing THA, such as hypertension (75% *versus* 55%) and diabetes (22% *versus* 11%). Roughly 17% of patients in the sample presented with at least one clinical risk for VTE.^1^

**Table 1:** Sociodemographic characteristics of the sample

Sociodemographic characteristics	TKA (n=121)	THA (n=94)	Total (n=215)
Mean age, mean (SD)	68.2 (9.8)	56.3 (15.6)	63.0 (14.0)
Sex, n (%)			
Male	30 (24.8)	44 (46.8)	74 (34.4)
Female	91 (75.2)	50 (53.2)	141 (65.6)
Smoking, n (%)			
Current	5 (4.1)	11 (11.7)	16 (7.4)
Prior	9 (7.4)	19 (20.2)	28 (13.0)
Mean BMI, SD	29.5 (5.8)	27.7 (4.5)	28.6 (5.3)
Hypertension, n (%)	90 (74.4)	52 (55.3)	142 (66.1)
*Diabetes mellitus*, n (%)	27 (22.3)	10 (10.6)	37 (17.2)
Clinical risk of VTE,* n (%)	21 (17.4)	15 (16.0)	36 (16.7)

TKA: total knee arthroplasty; THA: total hip arthroplasty; SD: standard deviation; BMI: body mass index; VTE: venous thromboembolism. *Report of at least one of the following: cancer, stroke, congestive heart failure, acute myocardial infarction, chronic venous or arterial insufficiency, atrial fibrillation, history of venous thromboembolism, bowel inflammatory disease, nephrotic syndrome, thrombophilia, bed-ridden or restricted to wheelchair, regular use of oral contraceptives or hormonal replacement therapy:^(1)^

In the SUS group, 87.7% (71/81) of patients received prophylaxis for VTE during hospitalization, and enoxaparin as the drug prescribed for all of them, regardless of the type of operation. In one patient submitted to TKA, enoxaparin was modified to non-fractioned heparin, with no report as to the reason for this. There was a report of use of compressive stockings as an adjuvant prophylactic method in only one patient. In this group, the scheme for administration of enoxaparin was similar to that of patients submitted to TKA and to THA, with a dosage of 40mg daily, initiating the medication, on average 29 hours after the surgical procedure and with a mean duration of 3 and 4 days during hospitalization, respectively. In the SS group, 98.5% (132/134) of patients performed some type of prophylaxis for VTE during hospitalization, with enoxaparin as the drug prescribed initially in 98.5% (130/132) of cases. For two patients (both submitted to THA), prophylaxis was done with dabigatran (110mg, once a day) to one, and compressive stockings for the other. In three patients, enoxaparin was replaced by dabigatran and in one patient, by non-fractioned heparin, with no report as to the reason. Among those who received prophylaxis, there was a report of use of compression stockings as an adjunctive prophylactic method in 16.7% (22/130), especially in the group submitted to TKA (22.8%). For most of the patients submitted to both TKA and THA, the dose of enoxaparin was 40mg daily and with a similar mean duration in hospitalization (4 days), although the mean time from the start of the medication after surgery was greater in the TKA group than in the THA group (20.5 hours *versus* 16.3 hours). Two patients received a scheme of enoxaparin 20mg daily, on average 7.8 hours after the surgical procedure with a mean duration of 5.5 days. A scheme of enoxaparin 60mg daily was given to two patients with a mean start of 50.5 hours after surgery and mean duration of 3.5 days during hospital stay.

Considering the total of 71 patients that received prophylaxis for VTE during their hospital stay, the total cost of prophylaxis for the SUS was R$ 1,873.01, generating a mean cost of R$ 26.38 per patient. Considering the total of 132 patients who received VTE prophylaxis during hospitalization, the total cost of prophylaxis for the SS was R$ 21,559.73, with a mean cost of R$163.33 per patient.

The incidence of VTE during hospitalization was 2.3% of suspected events and 0.9% of confirmed events. Among the patients submitted to TKA, the incidence of suspected or confirmed VTE was 1.6% (2/121) each; whereas for patients submitted to THA, only suspected events were observed, with an incidence of 3.2% (3/94).

The total cost and the mean cost per patient related to hospitalization (including the prophylaxis costs) are shown on table 2 for the patients seen by the SUS and by the SS. In the SUS group, the mean cost of hospital stay was R$ 6,210.80 per patient with suspected VTE and R$ 4,630.97 per patient without VTE. The mean length of hospital stay in these groups was 13 (SD=9.0) and 4.9 (SD=3.8) days, respectively. In the SS group, the mean cost of hospitalization was R$ 56,182.34 per patient with confirmed VTE, R$ 43,792.59 per patient with suspected VTE without confirmation, and of R$ 27,872.51 per patient with no event. The mean length of hospital stay was 22 (SD=7.1), 15.7 (SD=11.3), and 5.1 (SD=3.0) days in groups with confirmed VTE, suspected VTE, and without VTE, respectively.

**Table 2:** Patterns of prophylaxis, occurrence, and costs per segmentation in care of venous thromboembolism in the Unified Health System and Health Insurance System

	SUS		SS		Literature %				
	TKA %	THA %	TKA %	THA %					
Use of prophylaxis	88.0		98.5		13.24	38.71	38.92	86	80
					Surgical inpatients* (Engelhorn et al.^(16)^ Franco et al.^(17)^)	Orthopedics inpatients* (Engelhorn et al.^(16)^ Franco et al.^(17)^)	Patients in clinical and surgical wards (Deheinzelin et al.^(18)^)	Surgical patients in ICU (Carneiro et al.^(5)^)	Patients submitted to THA (Ramacciotti et al.^(19)^)
	
Type of prophylaxis									
Enoxaparin	100.0		98.6						
Dabigatran			0.8						
Enoxaparin replaced by dabigatran			2.3						
Adjuvant prophylaxis			17						
Compressive stockings			23	12					
Duration of prophylaxis (days)	Approximately 4								
Initiating medication after surgical procedure (hours)	29		20	16	enoxaparin 30mg SC every 12 hours, starting 12 to 24 hours before surgery, or 40mg SC per day, initiating 10 to 12 hours before surgery (Bastos^(3)^).				
Incidence of VTE after TKA or THA					2.4 a 40.4 depending on the type of surgery and diagnostic criteria (Cha et al.^(8)^; Clayton et al.^(9)^; Leizorovicz^(20)^)	8.8 of suspected VTE in THA, in which 10.3 had received drug prophylaxis and 2.6 had not (Ramacciotti et al.^(19)^)	Greater incidence of VTE in individuals submitted to TKA as compared to THA (Cha et al.^(8)^; Leizorovicz^(20)^)		
Occurrence of suspected VTE during hospitalization	2.5**		2.2***						
Length of hospital stay (days)									
With suspected VTE	13 (SD 9.0)		15.7 (SD 11.3)						
Without suspected VTE	4.9 (SD 3.8 )		5.1 (SD 3.0)						
Confirmed VTE	-		22 (SD 7.1)						
Mean estimated cost of hospital stay per patient (R$)									
With suspected VTE	6,210.80		43,792.59						
Without suspected VTE	4,630.97		27,872.51						
Confirmed VTE	-		56,182.34						
Total cost of prophylaxis for VTE (R$)	1,873.01		21,559.73						
Mean cost of prophylaxis per patient (R$)	26.38		163.33						

TKA: total knee arthroplasty; THA: total hip arthroplasty; ICU: intensive care unit; SC: subcutaneous; VTE: venous thromboembolism; SD: standard deviation; SUS: Unified Health System; SS: health insurance system. * At organizations financed by the SUS; **no case was confirmed; ***confirmation of VTE in 1.5% of operations.

## DISCUSSION

The present study evaluated how prophylaxis for VTE is conducted in patients submitted to TKA and THA operations within the Brazilian reality, in the context of both public and private health services. Additionally, it presented yet unpublished results as to the costs of prophylaxis for VTE and incremental expenses for the health system related to the occurrence of VTE during surgical hospitalization.

The use of prophylaxis for VTE was different in patients seen by the SUS and SS, even though the recommended regimen, as a whole, was similar. In the SUS, prophylaxis was not carried out in 12% of the patients (11% for TKA and 16% for THA) compared with 1.5% of patients (3% for TKA) seen at the SS. Most prior studies that investigated the use of prophylaxis for VTE used populations different from those assessed in our study, and were generally formed by general surgical patients or those hospitalized due to some clinical condition, demonstrating various results. Older studies, such as those by Engelhorn et al. and Franco et al., found results quite different relative to the present study. Only 13.24% of the surgical inpatients and 38.71% of orthopedics inpatients, respectively, in organizations financed by the SUS, received some type of VTE prophylaxis.^16,17^ A multicenter study carried out in other organizations of the State of São Paulo found a frequency of 38.92% of thromboprophylaxis for patients admitted to clinical and surgical wards. Carneiro et al.^5^ demonstrated that 86% of the surgical patient in an ICU received any type of prophylaxis for VTE. Only one study, performed in 2.000, evaluated patients submitted to THA in Brazil, demonstrating that 80% of patients were submitted to some type of drug prophylaxis.^19^

For those who received prophylaxis, enoxaparin was the medication prescribed in 100% and 98.6% in the SUS and in the SS, respectively. In the SS, dabigatran was prescribed for one (0.8%) patient and replaced enoxaparin in three (2.3%) patients, and compressive stockings were used as adjuvant prophylaxis in 17% of cases (23% of TKA and 12% of THA). The duration of prophylaxis during hospital stay was similar (about 4 days), initiating medication after the surgical procedure in all cases, but earlier at the SS (20 hours for TKA and 16 hours for THA) than at the SUS (29 hours for both surgeries).

According to Bastos et al.,^3^in an article published in a Brazilian journal summarizing the main recommendations for thromboprophylaxis in patients submitted to orthopedic surgery, the recommended scheme in this subgroup, considering the use of enoxaparin in most patients, would be enoxaparin 30mg subcutaneous every 12 hours, starting 12 to 24 hours before surgery, or 40mg subcutaneous daily, starting 10 to 12 hours before surgery.^3^ As was previously described, all patients in the sample analyzed initiated prophylaxis after the surgical procedure, and in a small proportion of the patients (2 to 12%), prophylaxis was not prescribed. Thus, it can be observed that compliance with national protocols was not complete as to the use of thromboprophylaxis in 100% of patients submitted to arthroplasty and when used, did not follow the recommendations as to time of start.^3^

The incidence of VTE among individuals submitted to TKA or THA described in the literature varied from 2.4 to 40.4%, depending on the type of surgery performed and the criteria used for the diagnosis.^20,21^ Ramacciotti et al.^19^ found an incidence of 8.8% of suspected VTE cases in individuals submitted to THA in 16 Brazilian health centers, and among them 10.3% had received drug prophylaxis and 2.6% had not been submitted to this treatment. Despite the findings demonstrated by this study corroborating those of the investigation by Ramacciotti et al.^19^ as to a smaller frequency of VTE in individuals who used prophylaxis, neither sample was calculated to test this hypothesis and most of the patients included received prophylaxis. Additionally, the studies were not designed to evaluate the efficacy of the use of prophylaxis. Similar to our results, two studies demonstrated that the incidence of VTE observed in individuals submitted to TKA was greater than that seen for the individuals submitted to THA.^8,20^

Among the patients seen by the SUS and analyzed in this study, the occurrence of VTE was suspected during the period of hospital stay in 2.5% of operations performed during that period (1.6% for TKA and 5.3% for THA), but no case was confirmed. The mean length of hospital stay was greater among the patients with suspected VTE, as well as the mean cost estimated for hospital stay. In the group of patients with suspected VTE, the length of the hospital stay was 13 (SD=9.0) days and the mean cost was R$ 6,210.80 per patient, while among those without suspected VTE, the length was 4.9 (SD=3.8) days, with a cost of R$ 4,630.97 per patient. Whereas the total cost of prophylaxis for VTE was R$ 1,873.01, with mean cost of R$ 26.38 per patient submitted to prophylaxis at the SUS.

Among the patients seen by the SS, the incidence of suspected VTE was 2.2% during hospital stay (1.7% for TKA and 2.7% for THA). The occurrence of VTE was confirmed in 1.5% of the operations, all of them TKA (one case of DVT and one case of DVT and PE). The mean length of stay and the mean estimated cost of the hospital stay were progressively greater for cases of suspected and confirmed VTE – in that, 22 (SD=7.1) days and R$ 56,182.34 per patient in the confirmed VTE group; 15.7 (SD=11.3) days and R$ 43,792.59 per patient in the suspected VTE group; and 5.1 (SD=3.0) days and R$ 27,872.51 per patient in the group with no events. The total cost of prophylaxis was R$ 21,559.73, with mean cost of R$ 163.33 per patient submitted to prophylaxis at the SS.

Thus, the incidence of 0.9% of VTE in the total sample of the study increased four-fold the length of hospital stay and duplicated the cost (R$ 28,309.83 per patient) for a private institution. Moreover, despite the small incidence, the length of hospital stay of patients with suspected VTE was equally increased in both health systems, even with greater cost for the private sector. Therefore, the inconsistency between the VTE prevention rates at hospitals and what is currently recommended creates concerns as to the understanding of the economic implications of VTE relative to healthcare costs, which have never been evaluated in Brazilian research. The results of this study demonstrate a cost per patient with confirmed VTE related to a longer hospital stay greater than the total cost of prophylaxis, reflecting that a case of VTE is more costly for the private sector than the 132 prophylaxis schemes. These estimates suggest, in an objective manner, how much could be saved by the Brazilian health service if prophylaxis regimens were used for all patients, theoretically avoiding one case of VTE.

Our study showed that, in the public service, most patients submitted to arthroplasty received thromboprophylaxis and benefited from it.

One of the limitations of this study was the fact of having compared only two private hospitals to one public hospital.

## CONCLUSION

The incidence of peripheral venous thrombosis in patients submitted to arthroplasty of the hip and knee is similar, with a moderate increase in absence of prophylaxis at the public hospital. In the next years, with the gradual introduction of new oral anticoagulants for prophylaxis on the market, it will be interesting to know how the public sector should position itself as to standardization of oral medications.
