# Supplementary material for: Comparison of thromboprophylaxis patterns in arthroplasty in public and private hospitals
Source: Einstein (Sao Paulo). 2015 Jul-Sep;13(3):410–6. doi: 10.1590/S1679-45082015GS3057 (PMC4943787; doi:10.1590/S1679-45082015GS3057)
Supplement: Translation

# Translation: Comparação entre padrões de tromboprofilaxia na artroplastia em hospitais público e privado

**Authors:** Aline Pinheiro dos Santos Cortada, Telma Gomes da Silva, André Campos da Silva, Ricardo Prado Golmia, Renata Leborato Guerra, Maíra Libertad Soligo Takemoto, Roberta Dyonisio Canaveira Monteiro, Morton Aaron Scheinberg

**Affiliations:** 1 Associação de Assistência à Criança Deficiente, São Paulo, SP, Brasil.; 2 Hospital Israelita Albert Einstein, São Paulo, SP, Brasil.; 3 ANOVA Consultoria em Saúde, Rio de Janeiro, RJ, Brasil.; 4 Bristol-Myers Squibb, São Paulo, SP, Brasil.

**Keywords:** Tromboembolia venosa/terapia, Artroplastia do joelho/economia, Artroplastia de quadril/economia, Sistema de saúde/economia, Hospitais públicos, Hospitais privados

## Abstract

**Objetivo:**

Comparar a terapia para profilaxia de tromboembolismo venoso e os custos de pacientes submetidos à artroplastia total de joelho e de quadril dentro do sistema de saúde brasileiro.

**Métodos:**

Estudo retrospectivo com pacientes submetidos à artroplastia no ano de 2010, em um hospital público e dois hospitais privados no Estado de São Paulo, por meio da revisão de prontuários. Os custos foram estimados com base na utilização de recursos em saúde durante a hospitalização. Análise descritiva de frequência e média (desvio padrão), de acordo com o tipo de atendimento em saúde (público ou privado).

**Resultados:**

Um total de 215 pacientes foram avaliados, sendo 56,3% submetidos à cirurgia de joelho e 43,7% à cirurgia de quadril. Cerca de 88% e 98% dos pacientes provenientes do serviço público e privado de saúde, respectivamente, receberam algum tipo de profilaxia para tromboembolismo, sendo a enoxaparina o medicamento mais utilizado em ambos sistemas. O custo total da profilaxia foi de R$ 1.873,01 (R$ 26,38 por paciente) no serviço público e R$ 21.559,73 (R$ 163,33 por paciente) no serviço privado. Para os indivíduos com tromboembolismo, o custo médio da internação foi de R$ 6.210,80 e R$ 43.792,59 por paciente atendido nos serviços de saúde público e privado, respectivamente.

**Conclusão:**

A profilaxia em pacientes submetidos à artroplastia é mais utilizada em pacientes do serviço de saúde privado do que público, apesar dos altos custos em ambos os serviços. Os pacientes com tromboembolismo tiveram um custo maior do que aqueles apenas com profilaxia, mostrando que a prevenção está associada a um maior custo-benefício.

## INTRODUÇÃO

O tromboembolismo venoso (TEV) é uma condição caracterizada por trombose venosa profunda (TVP) com a possibilidade de evolução para embolia pulmonar (EP).^(^ ^1^ ^,^ ^2^ ^)^ As cirurgias de artroplastia total de joelho (ATJ) e artroplastia total de quadril (ATQ) representam um importante fator associado à ocorrência de TEV, aumentando o risco de sua ocorrência em 40 a 70%. Nesses casos, é possível a adoção de medidas preventivas, o que torna o TEV a principal causa de óbito hospitalar evitável.

A tromboprofilaxia pode ser realizada com estratégias medicamentosas e não medicamentosas.^(^ ^3^ ^)^ Estudos demonstram que, em diversos países, inclusive no Brasil, a principal estratégia utilizada para tromboprofilaxia é a medicamentosa, sendo a heparina não fracionada e a heparina de baixo peso molecular os principais anticoagulantes utilizados.^(^ ^4^ ^)^ Entretanto, estudo realizado em um hospital de grande porte no Brasil, em 2007, avaliou como inadequada a conduta para tromboprofilaxia em 47% dos casos, quando comparada a 8ª Diretriz para Profilaxia de TEV do American College of Chest Physicians. Ausência de tromboprofilaxia foi a principal causa de inadequação.^(^ ^5^ ^)^

A ocorrência de TEV em pacientes submetidos a ATJ e ATQ pode apresentar reflexos diretos na morbidade dos pacientes e nos gastos com hospitalização. A TVP e a EP podem adiar a alta hospitalar em, aproximadamente, 5 e 7 dias, respectivamente, sendo a principal causa de readmissão hospitalar após ATQ.^(^ ^6^ ^)^ Nos Estados Unidos, um ano após apresentar um episódio de TEV, 5,3% dos pacientes necessitaram de reinternação hospitalar com TEV como causa primária e 14,3% como causa secundária.^(^ ^7^ ^)^

Dados sobre os padrões de profilaxia para TEV e custos associados ao cuidado dos pacientes submetidos à cirurgia de ATJ ou ATQ no Brasil são limitados.^(^ ^8^ ^-^ ^10^ ^)^ Este estudo foi desenvolvido com o intuito de preencher essa lacuna de dados locais. Dessa forma, o objetivo do estudo foi descrever os padrões de tromboprofilaxia de TEV e os custos durante o período de hospitalização de pacientes submetidos à ATJ e à ATQ dentro do contexto do sistema de saúde brasileiro.

## OBJETIVO

Comparar padrões de tromboprofilaxia nos sistema de saúde brasileiro público e privado.

## MÉTODOS

Estudo observacional em coorte retrospectiva, realizado em três hospitais, sendo dois representativos do sistema suplementar de saúde (Hospital da Associação de Assistência à Criança Deficiente − AACD e Hospital Santa Cruz, ambos na cidade de São Paulo − SP) e um do sistema público de saúde (Hospital Mário Covas da Faculdade de Medicina do ABC, em Santo André − SP). Foram incluídos no estudo pacientes com idade mínima de 18 anos submetidos à cirurgia eletiva de ATQ ou ATJ entre 1 de janeiro de 2010 e 31 de dezembro de 2010 e que não estivessem em uso prévio de terapia antitrombótica.

Informações sobre características sociodemográficas, clínicas e cirúrgicas dos pacientes, e sobre utilização de tromboprofilaxia foram coletadas de forma retrospectiva, a partir da revisão dos prontuários dos pacientes. Para fins deste estudo, TEV confirmado foi definido como relato, no prontuário, da confirmação de uma suspeita clínica do evento pelo médico, além de laudo positivo de algum método diagnóstico (ultrassonografia Doppler de vasos, tomografia computadorizada helicoidal e arteriografia pulmonar). Para a estimativa dos custos associados à hospitalização dos pacientes, foram coletadas informações sobre os tipos e a quantidade de recursos em saúde utilizados, como medicamentos, dispositivos médicos (próteses), exames complementares, diárias em enfermaria e em unidade de terapia intensiva (UTI), e outros tratamentos. Os valores unitários de cada tipo de recurso foram obtidos de acordo com a fonte pagadora, sendo utilizadas as seguintes fontes oficias de precificação de recursos em saúde: Banco de Preços em Saúde do Ministério da Saúde do Brasil, versão 2008;^(^ ^11^ ^) ^Lista de Conformidade da Câmara de Regulação do Mercado de Medicamentos (CMED) da Agência Nacional de Vigilância Sanitária (ANVISA);^(^ ^12^ ^)^ Sistema de Gerenciamento da Tabela de Procedimentos (SIGTAP);^(^ ^13^ ^)^ quarta edição da Classificação Brasileira Hierarquizada de Procedimentos Médicos (CBHPM);^(^ ^14^ ^)^ Programa de Estudos Avançados em Administração Hospitalar e de Sistemas de Saúde do Hospital das Clínicas da Faculdade de Medicina da Universidade de São Paulo e da Fundação Getúlio Vargas (PROAHSA 41 E 50).^(^ ^15^ ^)^

Foi realizada uma análise descritiva das características da amostra, sendo as variáveis quantitativas expressas por média e desvio padrão, como medida de dispersão, e as variáveis qualitativas, por frequências e porcentagens. O custo total e o custo médio por paciente foram estimados de acordo com a segmentação do cuidado recebido pelo paciente (procedimento cirúrgico e acompanhamento hospitalar - incluindo os custos da profilaxia para TEV durante o período de internação). Os resultados foram estratificados considerando o tipo de cirurgia realizada (ATJ e ATQ), o tipo de sistema de saúde (Sistema Único de Saúde − SUS e sistema suplementar de saúde − SS) e de acordo com a suspeita ou ocorrência de TEV. Todas as análises foram realizadas utilizando os programas estatísticos Stata, versão MP11, e o R Project, versão 2.13.1.

O estudo foi aprovado pelo Comitê de Ética em pesquisa da AACD número 44/2011.

## RESULTADOS

Um total de 283 pacientes foi submetido à ATJ ou ATQ nos centros selecionados, durante o ano de 2010. No entanto, os prontuários de 50 pacientes (17,7%) estavam indisponíveis para consulta. Dentre os pacientes com informações disponíveis, 92,3% (215/233) foram incluídos no estudo e 7,7% (18/233) foram excluídos por não preencherem os critérios de elegibilidade. Dentre os pacientes avaliados no estudo, 38% (81/215) eram provenientes do SUS e 62% (134/215) do sistema SS. Na figura 1, é apresentado o fluxograma da população do estudo.

**Figura 1:** Fluxograma da população do estudo

Cerca de 56% (121/215) dos pacientes da amostra foram submetidos à cirurgia de ATJ e 44% (94/215) à ATQ, sendo artroplastia primária em 80,2% (97/121) e 75,5% (71/94), e unilateral em 97,5% (118/121) e 100% (94/94) dos pacientes, respectivamente. A duração média da cirurgia de ATJ foi de 2 horas e 55 minutos, com média de internação de 5 dias; ao passo que, para ATQ, as médias foram, respectivamente, de 3 horas e 27 minutos, e de 6 dias. Maior frequência de sangramento como complicação foi observada na ATQ (11,7% *versus *1,7%).

As características da amostra estudada estão descritas na tabela 1. Os pacientes submetidos à ATJ apresentavam média de idade de 68,2 anos (DP=9,8) e cerca de 75% eram do sexo feminino, enquanto que pacientes submetidos à ATQ apresentavam média de 56,3 anos (DP=15,6) e 53% eram do sexo feminino. A maioria dos pacientes submetidos a ambas cirurgias não era tabagista, apresentava sobrepeso, com índice de massa corporal (IMC) médio de 29,5kg/m^2^ para ATJ e 27,7kg/m^2^ para ATQ. Os pacientes submetidos à ATJ tinham mais comorbidades que aqueles operados para ATQ, como hipertensão (75% *versus* 55%) e diabetes (22% *versus* 11%). Aproximadamente, 17% dos pacientes da amostra apresentavam pelo menos um risco clínico para TEV.^(^ ^1^ ^)^

**Tabela 1:** Características sociodemográficas da amostra

Características sociodemográficas	ATJ (n=121)	ATQ (n=94)	Total (n=215)
Média de idade, média (DP)	68,2 (9,8)	56,3 (15,6)	63,0 (14,0)
Sexo, n (%)			
Masculino	30 (24,8)	44 (46,8)	74 (34,4)
Feminino	91 (75,2)	50 (53,2)	141 (65,6)
Tabagismo, n (%)			
Atual	5 (4,1)	11 (11,7)	16 (7,4)
Pregresso	9 (7,4)	19 (20,2)	28 (13,0)
IMC média, DP	29,5 (5,8)	27,7 (4,5)	28,6 (5,3)
HAS, n (%)	90 (74,4)	52 (55,3)	142 (66,1)
*Diabetes mellitus*, n (%)	27 (22,3)	10 (10,6)	37 (17,2)
Risco clínico de TEV,* n (%)	21 (17,4)	15 (16,0)	36 (16,7)

ATJ: artroplastia total de joelho; ATQ: artroplastia total de quadril; DP: desvio padrão; IMC: índice de massa corporal; HAS: hipertensão arterial sistêmica; TEV: tromboembolismo venoso. *Relato de pelo menos um dos seguintes: câncer, derrame cerebral, insuficiência cardíaca congestiva, infarto agudo do miocárdio, insuficiência venosa ou arterial crônica, fibrilação atrial, história de tromboembolismo venoso, doença inflamatória intestinal, síndrome nefrótica, trombofilia, restrição ao leito ou cadeira de rodas, uso regular de contraceptivos orais ou terapia de reposição hormonal:^(1)^

No grupo do SUS, 87,7% (71/81) dos pacientes receberam profilaxia para TEV durante o período de internação, sendo a enoxaparina o medicamento prescrito para todos eles, independentemente do tipo de cirurgia. Em um paciente submetido à ATJ, a enoxaparina foi modificada para heparina não fracionada, sem relato de motivo. Houve relato do uso de meia compressiva como método profilático adjuvante para apenas um paciente. Nesse grupo, o esquema de administração da enoxaparina foi semelhante nos pacientes submetidos à ATJ e à ATQ, com a dosagem de 40mg ao dia, com início do medicamento, em média, 29 horas após o procedimento cirúrgico e duração média de 3 e 4 dias durante a internação, respectivamente. No grupo SS, 98,5% (132/134) dos pacientes realizaram algum tipo de profilaxia para TEV durante o período de internação, sendo a enoxaparina o medicamento prescrito inicialmente em 98,5% (130/132) dos casos. Para dois pacientes (ambos submetidos à ATQ), a profilaxia realizada foi dabigatrana (110mg, uma vez ao dia) para um e meia compressiva para o outro. Em três pacientes, a enoxaparina foi substituída por dabigatrana e, em um paciente, por heparina não fracionada, sem relato de motivo. Dentre os que receberam profilaxia, houve relato de meia compressiva como método profilático adjuvante em 16,7% (22/130), especialmente no grupo submetido à ATJ (22,8%). Para a maior parte dos pacientes, tanto aqueles submetidos à ATJ quanto à ATQ, a dosagem de enoxaparina foi de 40mg ao dia e com duração média, durante a internação, semelhante (4 dias), porém a média do tempo de início do medicamento após a cirurgia foi maior no grupo ATJ do que no grupo ATQ (20,5 horas *versus* 16,3 horas). Dois pacientes receberam esquema com enoxaparina 20mg ao dia, sendo iniciado, em média, 7,8 horas após o procedimento cirúrgico, com duração média de 5,5 dias. Esquema com enoxaparina 60mg ao dia foi administrado para dois pacientes com média de início de 50,5 horas após a cirurgia e duração média de 3,5 dias durante a internação.

Considerando o total de 71 pacientes que receberam profilaxia para TEV durante o período de internação, o custo total da profilaxia, para o SUS, foi de R$ 1.873,01, gerando um custo médio de R$ 26,38 por paciente. Considerando o total de 132 pacientes que receberam profilaxia para TEV durante o período de internação, o custo total da profilaxia para a SS foi de R$ 21.559,73, com custo médio de R$ 163,33 por paciente.

A incidência de TEV durante o período de internação hospitalar foi de 2,3% de eventos suspeitos e 0,9% de eventos confirmados. Entre os pacientes submetidos à ATJ, a incidência de TEV suspeito ou confirmado foi de 1,6% (2/121) cada. Entre os pacientes submetidos à ATQ, somente eventos suspeitos foram observados, com incidência de 3,2% (3/94).

O custo total e o custo médio por paciente relacionado à internação hospitalar (incluindo os custos da profilaxia) estão demonstrados na tabela 2 para pacientes atendidos pelo SUS e pela SS. No grupo SUS, o custo médio da internação foi de R$ 6.210,80 por paciente com suspeita de TEV e de R$ 4.630,97 por paciente sem TEV. A duração média da hospitalização nesses grupos foi de 13 (DP=9,0) e 4,9 (DP=3,8) dias, respectivamente. No grupo SS, o custo médio da internação foi de R$ 56.182,34 por paciente com TEV confirmado, R$ 43.792,59 por paciente com suspeita de TEV sem confirmação, e de R$ 27.872,51 por paciente sem evento. A duração média da internação foi de 22 (DP=7,1), 15,7 (DP=11,3) e 5,1 (DP=3,0) dias nos grupos com TEV confirmado, TEV suspeito e sem TEV, respectivamente.

**Tabela 2:** Padrões de profilaxia, ocorrência e custos por segmentação no cuidado de tromboembolismo venoso no Sistema Único de Saúde e sistema de saúde suplementar

	SUS		SS		Literatura %				
	ATJ %	ATQ %	ATJ %	ATQ %					
Uso de profilaxia	88,0		98,5		13.24	38.71	38,92	86	80
					Surgical inpatients* (Engelhorn et al.^(16) ^Franco et al.^(17)^)	Orthopedics inpatients* (Engelhorn et al.^(16)^ Franco et al.^(17)^)	Pacientes em alas clínicas e cirúrgicas (Deheinzelin et al.^(18)^)	Pacientes cirúrgicos em UTI (Carneiro et al.^(5)^)	Pacientes submetidos à ATQ (Ramacciotti et al.^(19)^)
Tipo de profilaxia									
Enoxaparina	100,0		98,6						
Dabigatrana			0,8						
Substituição da enoxaparina à dabigatrana			2,3						
Profilaxia adjuvante			17						
Meia compressiva			23	12					
Duração da profilaxia (dias)	Aproximadamente 4								
Início do medicamento após procedimento cirúrgico (horas)	29		20	16	enoxaparina 30mg SC a cada 12 horas, iniciado 12 a 24 horas antes da cirurgia, ou 40mg SC por dia, iniciado 10 a 12 horas antes da cirurgia (Bastos^(3)^).				
Incidência de TEV após ATJ ou ATQ					2,4 a 40,4 dependendo do tipo de cirurgia e critérios de diagnóstico (Cha et al.^(8)^; Clayton et al.^(9)^; Leizorovicz^(20)^)	8,8 de TEV suspeito em ATQ, sendo que 10,3 havia recebido profilaxia medicamentosa e 2,6 não (Ramacciotti et al.^(19)^)	Maior incidência de TEV em indivíduos submetidos à ATJ comparados à ATQ (Cha et al.^(8)^; Leizorovicz^(20)^)		
Ocorrência de TEV suspeito durante internação	2,5**		2,2***						
Duração da internação (dias)									
Com suspeita de TEV	13 (DP 9,0)		15,7 (DP 11,3)						
Sem suspeita de TEV	4,9 (DP 3,8)		5,1 (DP 3,0)						
TEV confirmado			22 (DP 7,1)						
Custo médio estimado da internação por paciente (R$)									
Com suspeita de TEV	6.210,80		43.792,59						
Sem suspeita de TEV	4.630,97		27.872,51						
TEV confirmado			56.182,34						
Custo total da profilaxia para TEV (R$)	1.873,01		21.559,73						
Custo médio da profilaxia por paciente (R$)	26,38		163,33						

ATJ: artroplastia total de joelho; ATQ: artroplastia total de quadril; UTI: unidade de terapia intensiva; SC: subcutâneo; TEV: tromboembolismo venoso; DP: desvio padrão; SUS: Sistema Único de Saúde; SS: sistema de saúde suplementar. * Em instituições financiadas pelo SUS; **nenhum caso foi confirmado; ***confirmação de TEV em 1,5% das cirurgias.

## DISCUSSÃO

O presente estudo avaliou como a profilaxia para TEV é realizada em pacientes submetidos à cirurgia de ATJ e ATQ na realidade brasileira, no âmbito de atendimento de saúde tanto público quanto privado. Além disso, apresentou resultados inéditos até o momento sobre os custos da profilaxia para TEV e custos incrementais para o sistema de saúde relacionados à ocorrência de TEV durante a internação cirúrgica.

A realização de profilaxia para TEV foi diferente em pacientes atendidos pelo SUS e pela SS, ainda que o esquema prescrito tenha sido, de forma geral, semelhante. No SUS, a profilaxia não foi realizada em 12% dos pacientes (11% para ATJ e 16% para ATQ) comparado com 1,5% dos pacientes (3% para ATJ) atendidos na SS. A maioria dos estudos prévios que investigaram o uso de profilaxia para TEV utilizou populações diferentes daquela avaliada no nosso estudo, sendo formada geralmente por pacientes cirúrgicos gerais ou hospitalizados por alguma condição clínica, demonstrando resultados variados. Estudos mais antigos, como os de Engelhorn et al. e Franco et al., encontraram resultados bastante divergentes em relação aos do presente estudo. Somente 13,24% de pacientes cirúrgicos internados e 38,71% de pacientes internados no setor de ortopedia, respectivamente, em instituições financiadas pelo SUS, receberam algum tipo de profilaxia para TEV.^(^ ^16^ ^,^ ^17^ ^)^ Estudo multicêntrico conduzido em outros centros do Estado de São Paulo encontrou frequência de 38,92% de tromboprofilaxia para pacientes internados em alas clínicas e cirúrgicas. Carneiro et al.^(^ ^5^ ^)^ demonstraram que 86% dos pacientes cirúrgicos internados em uma unidade de terapia intensiva receberam algum tipo de profilaxia para TEV Somente um estudo, realizado em 2,000, avaliou pacientes submetidos à ATQ no Brasil, demonstrando que 80% dos pacientes foram submetidos a algum tipo de profilaxia medicamentosa.^(^ ^19^ ^)^

Para aqueles que receberam profilaxia, enoxaparina foi o medicamento prescrito em 100% e 98,6% no SUS e na SS, respectivamente. Na SS, dabigatrana foi prescrito para um (0,8%) paciente e substituiu a enoxaparina em três (2,3%) pacientes, e meia compressiva foi utilizada como profilaxia adjuvante em 17% dos casos (23% de ATJ e 12% de ATQ). A duração da profilaxia durante a internação foi semelhante (cerca de 4 dias), com início do medicamento após o procedimento cirúrgico em todos os casos, porém mais precocemente na SS (20 horas para ATJ e 16 horas para ATQ) do que no SUS (29 horas para ambas).

De acordo com Bastos et al.,^(^ ^3^ ^)^ em artigo nacional que resume as principais recomendações para tromboprofilaxia em pacientes submetidos a cirurgia ortopédica, o esquema recomendado nesse subgrupo, assumindo uso de enoxaparina na maioria dos pacientes, seria enoxaparina 30mg subcutânea a cada 12 horas, iniciado 12 a 24 horas antes da cirurgia, ou 40mg subcutânea por dia, iniciado 10 a 12 horas antes da cirurgia.^(^ ^3^ ^)^ Conforme descrito anteriormente, todos os pacientes da amostra analisada iniciaram a profilaxia após o procedimento cirúrgico, sendo que, em pequena proporção dos pacientes (2 a 12%), a profilaxia não foi prescrita. Observa-se, portanto, que a adesão aos protocolos nacionais não foi completa, no que diz respeito à realização de tromboprofilaxia em 100% dos pacientes submetidos à artroplastia e, quando realizada, não obedeceu às recomendações quanto ao tempo de início.^(^ ^3^ ^)^

A incidência de TEV entre indivíduos submetidos à ATJ ou à ATQ descrita na literatura variou de 2,4 a 40,4%, dependendo do tipo de cirurgia realizada e dos critérios utilizados para o diagnóstico.^(^ ^20^ ^,^ ^21^ ^)^ Ramacciotti et al.^(^ ^19^ ^)^ encontraram incidência de 8,8% de TEV suspeito em indivíduos submetidos a ATQ em 16 centros de saúde brasileiro, entre eles 10,3% tinham recebido profilaxia medicamentosa e 2,6% não tinham sido submetidos a esse tratamento. Apesar dos achados demonstrados por este estudo corroborarem aqueles do estudo de Ramacciotti et al.^(^ ^19^ ^)^ quanto a uma menor frequência de TEV em indivíduos que utilizaram profilaxia, ambas as amostras não foram calculadas para testar essa hipótese e a maioria dos pacientes incluídos recebeu profilaxia. Além disso, os estudos não foram desenhados para avaliar a eficácia do uso da profilaxia. Semelhante aos nossos resultados, dois estudos demonstraram que a incidência de TEV observada em indivíduos submetidos à ATJ foi maior do que aquela observada para os indivíduos submetidos a ATQ.^(^ ^8^ ^,^ ^20^ ^)^

Entre pacientes atendidos no SUS analisados neste estudo, a ocorrência de TEV foi suspeita durante o período de internação hospitalar em 2,5% das cirurgias realizadas no período (1,6% para ATJ e 5,3% para ATQ), porém nenhum caso foi confirmado. A duração média da hospitalização foi maior entre os pacientes com suspeita de TEV, assim como o custo médio estimado da internação. No grupo de pacientes com suspeita de TEV, a duração da internação foi de 13 (DP=9,0) dias e o custo médio de R$ 6.210,80 por paciente, enquanto entre aqueles sem suspeita de TEV, esse tempo foi de 4,9 (DP=3,8) dias, com custo de R$ 4.630,97 por paciente. Já o custo total da profilaxia para TEV foi de R$ 1.873,01, com custo médio de R$ 26,38 por paciente submetido à profilaxia no SUS.

Entre pacientes atendidos na SS, a incidência de TEV suspeita foi de 2,2% durante a internação hospitalar (1,7% para ATJ e 2,7% para ATQ). A ocorrência de TEV foi confirmada em 1,5% das cirurgias, sendo todas de ATJ (um caso de TVP e um caso de TVP e EP). A duração média e o custo médio estimado da internação foram progressivamente maiores para casos de suspeita e de confirmação de TEV, sendo 22 (DP=7,1) dias e R$ 56.182,34 por paciente no grupo de TEV confirmado; 15,7 (DP=11,3) dias e R$ 43.792,59 por paciente no grupo da suspeita de TEV e 5,1 (DP=3,0) dias e R$ 27.872,51 por paciente no grupo sem evento. O custo total da profilaxia foi de R$ 21.559,73, com custo médio de R$ 163,33 por paciente submetido à profilaxia na SS.

Assim, a incidência de 0,9% de TEV na amostra total do estudo aumentou em quatro vezes a duração da internação hospitalar e duplicou seu custo (R$ 28.309,83 por paciente) para o serviço privado. Ainda, apesar de pequena incidência, a duração da internação hospitalar de pacientes com TEV suspeita foi igualmente aumentada em ambos os serviços de saúde, ainda que com maior custo para o setor privado. Dessa forma, a inconsistência entre as taxas de realização de prevenção para TEV nos hospitais e o que é atualmente recomendado levanta preocupações sobre a compreensão das implicações econômicas do TEV em relação aos custos de saúde, nunca antes avaliados em estudos nacionais. Os resultados desse estudo demonstram um custo por paciente com TEV confirmado relacionado à internação hospitalar maior do que o custo total da profilaxia, refletindo que um caso de TEV é mais custoso para o setor privado do que os 132 esquemas de profilaxia. Essas estimativas sugerem, de forma objetiva, o quanto poderia ser economizado pelo serviço de saúde brasileiro ao se utilizarem esquemas de profilaxia para todos os pacientes e, teoricamente, evitar um caso de TEV.

Nosso estudo mostrou que, no serviço público, a maioria dos pacientes submetidos à artroplastia recebe e se beneficia da tromboprofilaxia.

Uma das limitações deste estudo foi o fato de ter comparado somente dois hospitais privados com um público.

## CONCLUSÃO

A incidência de trombose venosa periférica em pacientes submetidos a artroplastia de quadril e joelho é semelhante com um moderado aumento na ausência de profilaxia no hospital público. Nos próximos anos, com a entrada gradual de novos anticoagulantes orais para profilaxia, será interessante saber como o setor público deverá se posicionar com relação à padronização dos medicamentos orais.
